# Molecular Detection of Arthropod-Borne Pathogens in Eurasian Badgers (*Meles meles*) from the United Kingdom

**DOI:** 10.3390/ani10030446

**Published:** 2020-03-06

**Authors:** Lisa Guardone, Valentina Virginia Ebani, Ranieri Verin, Simona Nardoni, Antonio Consolazione, Malcolm Bennett, Francesca Mancianti

**Affiliations:** 1 Department of Veterinary Sciences, University of Pisa, Viale delle Piagge 2, 56124 Pisa, Italy; lisa.guardone@for.unipi.it (L.G.); simona.nardoni@unipi.it (S.N.); a.consolazione987@gmail.com (A.C.); francesca.mancianti@unipi.it (F.M.); 2 Department of Comparative Biomedicine and Food Science, University of Padova, Viale dell’Università 16, 35020 Legnaro, Padova, Italy; ranieri.verin@unipd.it; 3 School of Veterinary Medicine and Science, University of Nottingham, Nottingham, LE12 5RD, UK; m.bennett@nottingham.ac.uk

**Keywords:** piroplasmida, *Babesia* sp., hemoprotozoa, arthropod-borne bacteria, wild carnivore, United Kingdom

## Abstract

**Simple Summary:**

Blood-sucking arthropods can cause infections in domestic and wild animals, as well as in humans by transmitting pathogens while blood feeding. During the first part of the twenty-first century, diseases caused by arthropods, including ticks, have increased significantly in Europe. In this study, the presence of different types of pathogens in badgers from Great Britain was evaluated by means of molecular techniques. While no bacteria were found, a blood parasite (*Babesia* sp.) was identified in half of the investigated animals. The same parasite has been reported in badgers from Spain, Scotland, China and Hungary, and recently also in a wild cat in Bosnia Herzegovina, a wolf in Italy and hunting dogs in Hungary, showing its widespread occurrence and potential presence in different host species. The impact on the health of these hosts and also of other wild carnivores needs to be further investigated.

**Abstract:**

Arthropod-borne diseases (ABD) are of increasing interest in veterinary and public health. Eurasian badgers (*Meles meles*) are known to harbor a wide range of pathogens, but information on their role as ABD reservoirs and their potential epidemiological relevance is limited. This study aimed to investigate the occurrence of arthropod-borne pathogens, specifically piroplasmids and the bacteria *Anaplasma phagocytophilum*, *Ehrlichia canis*, *Coxiella burnetii*, *Francisella tularensis* and *Bartonella* spp., in badgers from Great Britain (GB). Blood and heart samples from 18 badgers were examined using PCR and sequencing. A neighbour-joining (NJ) phylogram was also produced. Nine animals tested positive for *Babesia* sp., while none of the samples was positive for the investigated bacteria. The sequences obtained clustered with other sequences of *Babesia* sp. from badgers from GB and elsewhere, including China, Hungary, Spain and Italy, showing a widespread distribution of this parasite in badgers. Badger-associated *Babesia* DNA was also found recently in a wild cat in Bosnia Herzegovina, in a wolf in Italy and in dogs in Hungary. Further investigations are needed to understand the epidemiology of this putative pathogen and its impact on the health of wild and domestic carnivores.

## 1. Introduction

The incidence and distribution of arthropod-borne diseases have increased in Europe in recent decades as a result of increased awareness, global travel and trade and climatic and other environmental changes [1]. The main arthropod vectors threatening public health across Europe are mosquitoes, sandflies and ticks [2]. As an example, the UK reported the non-native mosquito *Aedes albopictus*, a known vector for dengue and chikungunya virus, in 2016. *Culex modestus*, the mosquito vector for the West Nile virus, has been detected since 2010. For tick-borne diseases, the changing distribution of *Ixodes ricinus*, the Lyme borreliosis vector, has been reported in parallel with an increase in the human reports of Lyme disease [3]. Among arthropod-borne diseases, tick-borne diseases (TBD) are particularly important to both human and veterinary medicine [4], and possibly to conservation biology. Several TBD have been reported in wildlife [5]. The adaptability of some species of wild carnivores to urban and peri-urban environments [6], and their role in the life cycle of pathogens of public health and veterinary interest is becoming increasingly evident [7].

The Eurasian (European) badger (*Meles meles*, Mustelidae family, Carnivora order) is widespread throughout Europe, and while it prefers mixed pasture and broadleaf woodlands, its generalist, highly adaptable nature enables it to exploit a wide variety of habitats [8,9]. Although relatively abundant in European countries, lower densities have been reported in some areas, sometime associated with hunting and “baiting”, and strong population fluctuations have been observed. This has prompted the implementation of conservation regulations and protected status since the 1970s and 1980s in several countries, such as the United Kingdom, Ireland, Spain, Portugal, Italy, Belgium, the Netherlands, Albania, Greece, Estonia, Luxemburg and Hungary [8]. The badger is Britain’s largest indigenous carnivore species and it has its own protection legislation, which is additional to general wildlife conservation legislation. Combined figures from England, Wales and Scotland suggest an estimated population of approximately 300,000 badgers [10].

Badgers may have an epidemiological role in infections of veterinary and public health importance, including the rabies virus, and most notably, bovine tuberculosis (*Mycobacterium bovis*), [11,12] as well as a range of other viruses, bacteria and endo- and ectoparasites [13]. Transmission, and therefore the veterinary and medical significance, may be enhanced in the case of relatively high population densities [9]. In the past decades, similarly to red foxes [14], populations of badgers have increased and adapted to peri-urban and urban environments across Europe [15], thus representing a possible source of pathogens for domestic animals and humans [16].

Among relevant arthropod-borne pathogens are *Babesia*, *Cytauxzoon* and *Theileria* species, all related hemoprotozoans collectively known as piroplasmids or piroplasms. Their life cycle is indirect; asexual reproduction occurs inside erythrocytes in the vertebrate hosts while the sexual phase takes place in ixodid ticks, which ingest and subsequently transmit the parasites while taking a blood meal [17]. An especially wide vertebrate host range is known for *Babesia* species, such that all vertebrate species may be susceptible to infection as long as they are also a host for *Babesia*-vector ticks. Various ticks have been reported as piroplasmid vectors. For *Babesia* and *Theileria* the most important genera are *Rhipicephalus*, *Dermacentor*, *Haemaphysalis*, *Boophilus*, *Ixodes* and *Hyalomma*, depending on the host species and the geographical area [17]. *Cytauxzoon* is mainly transmitted by *Dermacentor* and *Amblyomma* in North America, while its vector remains unknown in Europe [18].

Other tick-borne infections reported in Europe include *Ehrlichia canis*, which infects dogs, cats and wild canids and is usually transmitted by the brown dog-tick *Rhipicephalus sanguineus*, although other ticks may be involved in the transmission [19]. *Anaplasma phagocytophilum* is responsible for disease mainly in humans, horses and dogs, but it may also infect several wild mammal species and it is largely transmitted by ixodid ticks [20]. *Coxiella burnetii* may be transmitted by various tick species, although humans and animals usually acquire the infection through ingestion of raw milk and dairy products or inhalation of contaminated aerosols [21]. *Francisella tularensis* is able to infect several species of mammals, mainly lagomorphs, and is transmitted through hematophagous arthropods, but also through contact with infected animals as well as by drinking contaminated water [22], although it has never been reported in Great Britain. Finally, some *Bartonella* species may also be transmitted by ticks, as well as by other hematophagous arthropods [23], although tick competence is debated [24]. Bartonellae may infect domestic animals, mainly dogs and cats, even though wild mammals are frequently involved in the epidemiology of these agents [25].

This study investigated the occurrence of piroplasmids and arthropod-borne bacteria *Anaplasma phagocytophilum*, *Ehrlichia canis*, *Coxiella burnetii*, *Francisella tularensis* and *Bartonella* spp. in Eurasian badgers from Northwest England (GB).

## 2. Materials and Methods

### 2.1. Sampling and Post Mortem Examination

Eighteen carcasses of Eurasian badgers (8 males and 10 females), of different ages, were collected between 2016 and 2017 from Cheshire (Northwest England), United Kingdom. In all cases, the cause of death was attributed to road traffic collision. Carcass collection and sampling was carried out as part of a broader national project, focused on the prevalence of bovine tuberculosis in badgers.

The study had formal ethical approval from both the University of Liverpool and the University of Nottingham.

Ticks were not collected, as soon after the death of the host the majority of the ectoparasites drop off the hosts, making ticks generally not evaluable at post mortem examination.

A full post mortem examination was carried out on each subject within 24 h of receiving the carcasses. In the meantime, these were stored at 4 °C. Sex and age (juveniles or adults) of each carcass was recorded and based on body mass and dental wear as previously described [12]. Both blood and heart samples were collected from eight of the badgers, while for 10 animals only the heart was available (Table 1).

### 2.2. Molecular Analysis

Total DNA was extracted from blood (200 µL) and heart (10 mg) samples using the DNeasy Blood and Tissue Kit (QIAGEN), following the manufacturer’s blood and tissue protocols, respectively.

The total DNA obtained was submitted to different PCR protocols, targeting different gene fragments and using different primer pairs to screen for DNA of piroplasmids, *A. phagocytophilum*, *E. canis*, *C. burnetii*, *F. tularensis* and *Bartonella* spp. (Supplementary Materials, Table S1). For some pathogens, such as *A. phagocytophilum* and *E. canis,* a nested PCR protocol was used. PCR amplifications were performed using Wonder Taq (Euroclone, Milano, Italy) and an automated thermal cycler (Gene-Amp PCR System 2700, Perkin Elmer, Norwalk, CT, USA). Sterile distilled water instead of DNA was used as negative control. DNA extracted from slides for indirect immunofluorescent assay coated with *A. phagocytophilum*, *E. canis*, *C. burnetii*, *F. tularensis* and *Bartonella henselae* (Fuller Laboratories, Fullerton, CA, USA), and a known positive sample of *B. annae* previously sequenced [26] were included as positive controls.

PCR products were analysed by electrophoresis on 2% agarose gel stained with GelRed^®^ Nucleic Acid Gel Stain (Biotium). SharpMass™ 100 Plus Ladder (Euroclone, Milano, Italy) was used as a DNA marker. PCR products of the expected length and with a sufficient concentration were forward and reverse Sanger sequenced by an external company. Nucleotide sequences were analysed using Bioedit version 7.0.9 [27]. Adjustments were made after visual checking and consensus sequences were compared against those deposited in GenBank by using the National Center for Biotechnology Information (NCBI) Basic Local Alignment Search Tool (BLAST). A neighbour-joining (NJ) phylogram was constructed using Bioedit and MEGA-X software.

## 3. Results

At post mortem examination all the subjects showed good to fair body condition with adequate adipose tissue deposits (Table 1). No gross lesions, including lesions suggestive of bovine tuberculosis, other than road traffic related injuries, were observed.

DNA of arthropod-borne bacteria was not detected in any sample. DNA of piroplasmids was amplified in seven out of the eight blood samples examined, as well as in two additional heart samples (Table 1). Thus, overall, piroplasmid DNA was successfully amplified in 50% of the sampled badgers. Seven amplicons from blood samples were sequenced (the positive heart samples could not be sequenced due to their low concentration). All positive samples were identified as *Babesia* sp. In fact, all the sequences produced in the present study showed 100% identity values with sequences of *Babesia* sp. “type A” isolated from a badger in China and in Spain [16], as well as with *Babesia* sp. isolated from *Felis silvetris* in Bosnia and Herzegovina [28]. Slightly lower identity values (99.81–99.79%) were observed with sequences of *Babesia* sp. “type A” isolated from badgers in the UK (Scotland) [10]. Details of the BLAST results, including the accession number of the sequences with the highest identity values are reported in Supplementary Materials, Table S2.

**Figure 1:**
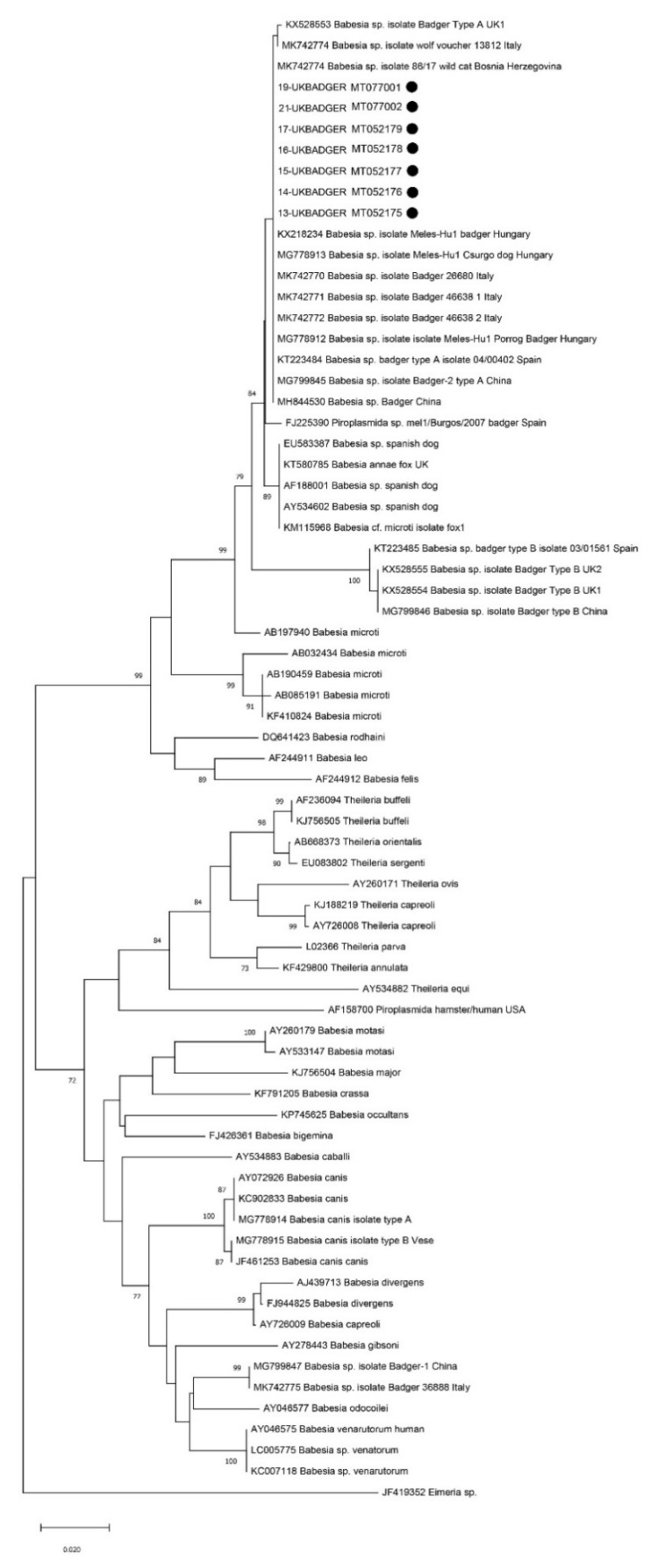
Neighbour-joining phylogram created with 7 sequences of the small subunit (18S) ribosomal RNA gene obtained in the present survey, 19 sequences of badger-associated *Babesia* sp. and 44 other piroplasmids sequences included in the phylogenetic analysis in recent works on *Babesia* sp. in badgers [9,10].

The NJ phylogram was constructed by aligning and trimming (to a final length of 455 bp) the seven sequences of the small subunit (18S) ribosomal RNA gene obtained in the present study, 19 sequences of badger-associated *Babesia* sp. and 44 other piroplasmids sequences already included in the phylogenetic analysis in recent works on *Babesia* in badgers [9,10]. All the sequences obtained in the present study clustered together, along with other *Babesia* sp. isolated from badgers from the UK (*n* = 1, deposited as “type A”), Italy (*n* = 3), Hungary (*n* = 2), Spain (*n* = 2, one of which deposited as “type A”) and China (*n* = 2, deposited as “type A”). Moreover, badger-associated *Babesia* sp. isolated from a wolf in Italy, from a wild cat in Bosnia Herzegovina and from a dog in Hungary also clustered in this group. All these sequences clustered separately from another cluster including four sequences from badgers (one from Spain, two from the UK and one from China) described as “type B” (Figure 1).

## 4. Discussion

None of the samples tested contained PCR-detectable *A. phagocytophilum*, *E. canis*, *F. tularensis*, *C. burnetii*, or *Bartonella* spp. As the number of samples tested was very small, any conclusion drawn from these results should be tentative. However, it is not surprising that *E. canis* and *F. tularensis* infection were not found as *E. canis* in British dogs has so far been largely (although not completely) limited to those with a history of travel to mainland Europe [29], and *F. tularensis* has never been reported in Great Britain, Ireland or Iceland [30]. A novel *Ehrlichia* sp. was found in a badger in Northern Spain [31] and isolates recently obtained from Italian badgers showed 99% similarity with it [32]. *C. burnetii* infection is endemic in British livestock, and seropositivity is common in people working with them. Infection often occurs without any obvious signs of disease in both animals and humans [33,34,35]. The role of wildlife in the epidemiology/ecology of Q-fever is unclear globally [36] and unknown in Great Britain, but the fact that the badgers in this study came from one of the densest dairy farming areas of Great Britain and none were infected suggests that they probably do not play a role in the ecology of the infection. *A. phagocytophilum* and *Bartonella* spp. infections are both found in domestic animals and wildlife in Great Britain and *A. phagocytophilum* was detected in 0.74% of ixodid ticks collected from British dogs [37]. However, as in this study, *A. phagocytophilum* was not detected in badgers in The Netherlands, Czech Republic, and Spain and only a low prevalence was observed among badgers in another study conducted in Belgium and The Netherlands, as well as in Italy, suggesting badgers do not play a significant epidemiological role in this infection ([32] and references therein). A previous survey found what appeared to be a novel *Bartonella* sp., most closely related to *B. clarridgeiae*, in 12% of badgers in northern Spain [38]. *B. clarridgeiae* infection is largely associated with cats and dogs, and more recently humans [39], so further investigation of a larger number of badgers would be interesting.

Tick-borne hemoparasites of the genus *Babesia* are reported to be the second most commonly found parasites in the blood of mammals after trypanosomes. Infection has been reported globally in a great variety of wild mammal species and some birds [17,40,41], as well as in badgers and other wildlife [5,16,26,42,43]. In recent reports, *Babesia* parasites have been molecularly characterised and the species’ descriptions in older studies have been questioned. *Babesia* from wildlife hosts may be distinguished as two types: parasites largely specific to a particular host, and babesias typically found in closely related domestic animals, e.g., *B. bigemina* in gazelles or *B. canis* in wolves [17]. According to the most recent molecular studies, *Babesia* spp. of carnivores can be divided into three groups: (i) the *B. microti* group infecting felids, canids, mustelids and procyonids; (ii) the prototheilerid group infecting felids, canids, herpestids and hyaenids; and (iii) the *Babesia sensu stricto* group infecting canids, procyonids, and ursids [44].

*Babesia* epidemiology in badgers has recently attracted the interest of research groups in various countries, as shown by the increasing number of publications in which variable prevalence values have been reported (Table 2). Although only a small number of badgers were tested in this study, the prevalence found is in broad agreement with the 70% prevalence found in 64 badgers sampled from East/Central Scotland. Prior to the work of Bartley et al. [10], which describes the first molecular identification of *Babesia* DNA from badgers in Britain, piroplasmosis were reported in this host in southwest England [45]. However, only low parasitemia of intraerythrocytic organisms resembling piroplasms were reported and these were not specifically identified. The vectors are likely *Ixodes canisuga* and *I. hexagonus*, badger ticks that are common in the area [46]. A subsequent study tested a total of 718 blood samples from 263 individual badgers over a three-year period, and found 77.2% individuals positive for *B. missirolii* (based on morphology) at least once over 3 years [47].

There are few reports of babesiosis in badgers from other countries, and they often describe only small-scale surveys (Table 2). As mentioned, the interest seems to have increased in the last decade following the report of a new piroplasmid in the Eurasian badger in Spain (accession number FJ225390) [42]. The species was shown to be distantly related to both *Theileria/Babesia annae* and a piroplasmid from *Lontra canadensis* and is considered as belonging to the group of the *Babesia microti*-like organisms found in the dog and small carnivores. Subsequent case reports and epidemiological studies reported the same type (named *Babesia* sp. “type A” by some authors) as well as different sequence types (including the genotype called *Babesia* sp. “type B” in some studies) occurring in other countries (Table 2). In particular, studies performed in Spain confirmed the presence of *Babesia* in badgers in that country [16]. Lately, *Babesia* sp. was identified in badgers in Hungary [9,44], Korea (although in this case without molecular investigation) [48] and very recently also in Italy [32,49]. In addition, a few *Babesia* sequences not referred to in any publication are also deposited in GenBank. These are derived from a badger from Xinjiang, China (*Babesia* sp. badger isolate Badger-1; MG799847.1) and from *I. canisuga* ticks collected from a red fox in Germany (Babesia sp. 4 NAN2012; JX679177.1).

Badger-associated *Babesia* sp. DNA has also been amplified from the blood of one European wild cat (*Felis silvestris*) in Bosnia and Herzegovina. This record, together with the finding of an *Hepatozoon* sp. type typical of the badger in *F. silvestris*, has been attributed to the sharing of burrows between badgers and other animals, which favours transmission of the potential vectors, especially nidicolous arthropods [28]. Additional epidemiological information may be obtained by investigating ticks rather than just vertebrate hosts. In Spain, a recent study found *B. annae/T. annae* in a pool of nymphs of *I. canisuga* from a badger, despite the badger not being detectably infected [7].

The high similarity of “type A” sequences obtained from Europe and China suggests this is a badger-adapted species/genotype [49]. Moreover, the finding of badger-associated *Babesia* sp. “type A” in a wild cat in Bosnia and Herzegovina [28], and recently in a grey wolf in Italy [49] and in six dogs in Hungary, one with relevant clinical signs [44], demonstrates the possibility of infection of a broad range of carnivore hosts and highlights the circulation of these species within the order Caniformia [32]. Walking in woodlands and hunting activities were identified as risk factors for dogs as these are associated with infestation with ticks [44].

## 5. Conclusions

This study confirms the widespread occurrence of badger-associated *Babesia* sp. (type A). As this and previous studies have mainly investigated a fragment of the 18 rRNA gene, further analysis of additional gene targets would be informative. Moreover, recent reports of the same badger-associated *Babesia* species/genotype in a wild cat, wolf and in clinically affected domestic dogs underlines the need for larger scale molecular surveys to investigate the potential risk posed by this putative pathogen for other wild carnivores as well as for domestic animals.

Even though the obtained results suggest that *M. meles* is not pivotal in the epidemiology of the other investigated arthropod-borne pathogens in the examined area, further investigation of a larger number of badgers could be useful to better understand their role in these infections.

## Figures and Tables

**Table 1:** Details of the sampled Eurasian badgers: sex, age class, weight, body condition score (BCS), status of the carcass at the time of sampling, availability of different tissues and results of the PCR for amplification of piroplasmid DNA. n.a.: not available.

N	Sex	Age Class	Weight (kg)	BCS	Carcass Status	PCR—Piroplasmid Blood Samples	PCR—Piroplasmid Heart Samples
1	F	Young	7.6	4/5	Good	n. a.	Neg
2	F	Adult	11.6	4/5	Good	n. a.	Neg
3	F	Adult	12.8	3/5	Good	n. a.	Pos
4	M	Adult	12	3/5	Mildly autolytic	n. a.	Pos
5	F	Juvenile	6.7	4/5	Moderately autolytic	n. a.	Neg
6	F	Adult	12.8	4/5	Good	n. a.	Neg
7	F	Adult	14.1	4/5	Good	n. a.	Neg
8	M	Adult	15.8	3/5	Autolytic	n. a.	Neg
9	M	Adult	10.1	4/5	Good	n. a.	Neg
10	F	Adult	14.3	3/5	Autolytic	n. a.	Neg
11	M	Adult	10.3	4/5	Good	Pos	Pos
12	M	Juvenile	10.5	3/5	Very good	Pos	Pos
13	M	Juvenile	9.8	3/5	Good	Pos	Pos
14	M	Adult	11.4	3/5	Good	Pos	Pos
15	F	Juvenile	9.4	3/5	Good	Pos	Pos
16	F	Adult	9.4	4/5	Good	Neg	Neg
17	F	Adult	11.6	3/5	Good	Pos	Pos
18	M	Juvenile	8.3	2/5	Autolytic	Pos	Pos

**Table 2:** Literature studies (since 2009) that report badger-associated *Babesia* sp. in Eurasian badgers (*Meles meles*) or in other carnivore species or ticks.

References	Geographical Area	Species	N	Positive/Examined (Prevalence %)	*Babesia* sp. Found
Battisti et al. 2020 [32]	Italy	Badger (*M. meles*)	45	41/45 (91.1%)	Badger-associated *Babesia* spp. type A and B; *B. capreoli, Babesia sp. DO23163*
Santoro et al. 2019 [49]	Italy	Badger (*M. meles*)	13	7/13 (53.8%)	Two sequence types: badger-associated *Babesia* spp. from MK742770 to MK742773 and MK742775
		Wolf (*Canis lupus*)	13	1/13 (7.7%)	Badger-associated *Babesia* spp. (MK742774)
Hodžić et al. 2018 [28]	Bosnia and Herzegovina	European wild cat (*Felis silvestris silvestris*)	18	5.5%	Badger-associated *Babesia* sp. (MF614153)
Bartley et al. 2017 [10]	UK (Scotland)	Badger (*M. meles*)	64	overall: 70.2%; Type A: 12·8% spleen and 38.3% blood; Type B: 19.1% spleen and 46.8% blood. Both type A and B sequences 10.6% spleen and 31.9% blood	*Babesia* Badger type A UK1 (KX528553), Badger type B UK1 (KX528554) and Badger type B UK2 (KX528555) additional sequences registered under accession nr. From KY250472 to KY250477 *
Hornok et al. 2017 [9]	Hungary	Badger (*M. meles*)	1	case report (*n* = 1)	*Babesia* sp. Meles-Hu1 (KX218234)
		*Ixodes rugicollis*	3	1/3 (33.3%)	*Babesia* sp. Meles-Hu1
Hornok et al. 2018 [44]	Hungary	Badger (*M. meles*)	5	5/5 (100%);	*Babesia* sp. Meles-Hu1 (MG778912)
		Dogs	90	6/90 (6.7%);	*Babesia* sp. Meles-Hu1 (MG778913)
		*Ixodes canisuga*	27	18/27 (66.7%)	*Babesia* sp. Meles-Hu1
Hong et al. 2017 [48]	Korea	Badgers	3	2/3 (66.7%)	*Babesia microti*
Barandika et al. 2016 [16]	Spain	Badger (*M. meles*)	122	64/122 (52.2%)	*Babesia* sp. badger type A (KT223484) and B (KT223485)
Millán et al. 2016 [7]	Spain	Badger (*M. meles*)	3	0	-
Gimenez et al. 2009 [43]	Spain	Badger (*M. meles*)	5	1/5 (20%)	Piroplasmida sp. mel1/Burgos/2007 (FJ225390)

* These sequences were not included in the NJ dendrogram as they only overlap the sequences obtained in the present study for approximately 220 bp.

## Supplementary Materials

The following are available online at https://www.mdpi.com/2076-2615/10/3/446/s1, Table S1: Gene targets, amplicon length, primer pairs and PCR conditions used for the different pathogens, Table S2: Details of the results of the BLAST analysis (https://blast.ncbi.nlm.nih.gov/Blast.cgi) for the samples PCR positive for piroplasmids.
