# Supplementary material for: Molecular Detection of Arthropod-Borne Pathogens in Eurasian Badgers (Meles meles) from the United Kingdom
Source: Animals (Basel). 2020 Mar 6;10(3):446. doi: 10.3390/ani10030446 (PMC7143893; doi:10.3390/ani10030446)
Supplement: Supplementary file 1 [file animals-10-00446-s001.pdf]

animals

Supplementary Materials: Molecular Detection of Arthropod-Borne Pathogens in Eurasian Badgers (*Meles meles*) from the United Kingdom

Lisa Guardone [1], Valentina Virginia Ebani [1,*], Ranieri Verin [2], Simona Nardoni [1], Antonio Consolazione [1], Malcolm Bennett [3] and Francesca Mancianti [1]

[1] Department of Veterinary Sciences, University of Pisa, Viale delle Piagge 2, 56124 Pisa, Italy; lisa.guardone@for.unipi.it (L.G.); simona.nardoni@unipi.it (S.N.); a.consolazione987@gmail.com (A.C.); francesca.mancianti@unipi.it (F.M.)
[2] Department of Comparative Biomedicine and Food Science, University of Padova, Viale dell'Università 16, 35020 Legnaro, Padova, Italy; ranieri.verin@unipd.it
[3] School Veterinary Medicine and Science, University of Nottingham, Nottingham, LE12 5RD, UK; m.bennett@nottingham.ac.uk
* Correspondence: valentina.virginia.ebani@unipi.it

Table S1. Gene target, amplicon length, primer pairs and PCR conditions used for the different pathogens.

Pathogen	Gene Target and Amplicon Length (bp)	Primers' Name and Sequence	PCR Conditions	References
Piroplasmid	18S rRNA [1] 560 bp	Mic1 GTCTTGTAATTGGAATGATGG Mic2 CCAAAGACTTTGATTTCTCTC	94 °C—30″ 50 °C—30″ 72 °C—1′	[1]
Anaplasma phagocytophilum	16S rRNA[2] 932 bp (First PCR)	GE3a CACATGCAAGTCGAACGGATTATTC GE10r TTCCGTTAAGAAGGATCTAATCTCC	95 °C—30″ 55 °C—30″ 72 °C—1′	[2]
	16S rRNA [2] 546 bp (Second PCR)	GE9f AACGGATTATTCTTTATAGCTTGCT GE2 GGCAGTATTAAAAGCAGCTCAGGG	95 °C—30″ 55 °C—30″ 72 °C—1′	
Ehrlichia canis	16S rRNA [2] 152 bp (First PCR)	ECCf AGAACGAACGCTGGCGGCAAGC ECBr CGTATTACCGCGGCTGCTGGCA	94 °C—1′ 55 °C—2′ 72 °C—1.5′	[3]
	16S rRNA [2] 395bp (Second PCR)	ECAN5 CAATTATTTATAGCCTCTGGCTATAGGA HE3r TATAGGTACCGTCATTATCTTCCCTAT	92 °C—1′ 55 °C—2′ 72 °C—1.5′	
Coxiella burnetii	IS1111a [3] 687 bp	TRANS-1 TATGTATCCACCGTAGCCAGT TRANS-2 CCCAACAACACCTCCTTATTC	95 °C—30″ 64 °C—1′ 72 °C—1′	[4]
Francisella tularensis	TUL4 [4] 400 bp	TUL4–435 TCGAAGACGATCAGATACCGTCG TUL4–863 TGCCTTAAACTTCCTTGCGAT	96 °C—1′ 60.5 °C—1′ 72 °C—1′	[5]
Bartonella spp.	16S rRNA [2] 296 bp	P24E CCTCCTTCAGTTAGGCTGG P12B GAGATGGCTTTTGGAGATTA	95 °C—1′ 57 °C—1′ 72 °C—1′	[6]

[1] 18S rRNA: 18S ribosomal RNA; [2] 16S rRNA: 16S ribosomal RNA; [3] ISS1111: Insertion Sequence; [4] TUL4: Tul4 membrane protein.

Table S2. Details of the results of the BLAST analysis (https://blast.ncbi.nlm.nih.gov/Blast.cgi) for the samples PCR positive for piroplasmids

Badger Code	Sequence Lenght (bp)	% Identity/ % Query Coverage	Description	Sequence Accession Nr	Origin	Species and Sample
UKBADGER13	520	100/100	*Babesia* sp. badger type A isolate Badger-2 18S rRNA gene	MG799845.1	China: Xinjiang	*M. meles*, blood
		100/100	*Babesia* sp. badger type A isolate 04/00402 18S rRNA gene	KT223484.1	Spain	*M. meles*, blood
		99.81/100	*Babesia* sp. isolate Badger Type A UK1 18S rRNA gene	KX528553.1	United Kingdom	*M. meles*, blood
		99.23/100	Piroplasmida sp. mel1/Burgos/2007 18S rRNA gene	FJ225390.1	Spain: Burgos	*M. meles*, blood
		100/97	*Babesia* sp. isolate 86/17 18S rRNA gene	MF614153.1	Bosnia—Herzegovina	*Felis silvestris*, spleen
UKBADGER14	473	100/100	*Babesia* sp. badger type A isolate Badger-2 18S rRNA gene	MG799845.1	China: Xinjiang	*M. meles* blood
		100/100	*Babesia* sp. isolate 86/17 18S rRNA gene	MF614153.1	Bosnia—Herzegovina	*Felis silvestris* spleen
		100/100	*Babesia* sp. badger type A isolate 04/00402 18S rRNA gene	KT223484.1	Spain	*M. meles* blood
		99.79/100	*Babesia* sp. isolate Badger Type A UK1 18S rRNA gene	KX528553.1	United Kingdom	*M. meles* blood
		100/99	*Babesia* sp. voucher 46638_2 small subunit ribosomal RNA gene	MK742772	Italy	*M. meles*, spleen
UKBADGER15	460	100/100	*Babesia* sp. badger type A isolate Badger-2 18S rRNA gene	MG799845.1	China Xinjiang	*M. meles* blood
		100/100	*Babesia* sp. isolate 86/17 18S rRNA gene	MF614153.1	Bosnia—Herzegovina	*Felis silvestris* speen
		100/100	*Babesia* sp. badger type A isolate 04/00402 18S rRNA gene	KT223484.1	Spain	*M. meles* blood
		99.78/100	*Babesia* sp. isolate Badger Type A UK1 18S rRNA gene	KX528553.1	United Kingdom	*M. meles* blood
		100/99	*Babesia* sp. voucher 46638_2 small subunit ribosomal RNA gene	MK742772	Italy	*M. meles*, spleen

UKBADGER16	481	100/100	*Babesia* sp. badger type A isolate Badger-2 18S rRNA gene	MG799845.1	China: Xinjiang	*M. meles* blood
		100/100	*Babesia* sp. isolate 86/17 18S rRNA gene	MF614153.1	Bosnia—Herzegovina	*Felis silvestris* speen
		100/100	*Babesia* sp. badger type A isolate 04/00402 18S rRNA gene	KT223484.1	Spain	*M. meles* blood
		99.79/100	*Babesia* sp. isolate Badger Type A UK1 18S rRNA gene	KX528553.1	United Kingdom	*M. meles* blood
		100/98	*Babesia* sp. voucher 46638_2 small subunit ribosomal RNA gene	MK742772	Italy	*M. meles*, spleen
UKBADGER17—19–21	531	100/100	*Babesia* sp. badger type A isolate Badger-2 18S rRNA gene	MG799845.1	China: Xinjiang	*M. meles* blood
		100/100	*Babesia* sp. badger type A isolate 04/00402 18S rRNA gene	KT223484.1	Spain	*M. meles* blood
		99.81/100	*Babesia* sp. isolate Badger Type A UK1 18S rRNA gene	KX528553.1	United Kingdom	*M. meles* blood
		99.25/100	Piroplasmida sp. mel1/Burgos/2007 18S rRNA gene	FJ225390.1	Spain: Burgos	*M. meles* blood
		100/97	*Babesia* sp. isolate 86/17 18S rRNA gene	MF614153.1	Bosnia—Herzegovina	*Felis silvestris* speen

Reference

1. Beck, R.; Vojta, L.; Mrljak, V.; Marinculić, A.; Beck, A.; Živičnjak, T.; Cacciò, S.M. Diversity of *Babesia* and *Theileria* species in symptomatic and asymptomatic dogs in Croatia. *Int J Parasitol* **2009**, *39*, 843–848. DOI: 10.1016/j.ijpara.2008.12.005
2. Massung, R.F.; Slater, K.; Owens, J.H.; Nicholson, W.L.; Mather, T.N.; Solberg, V.B., Olson, J.G. Nested PCR assay for detection of granulocytic ehrlichiae. *J Clin Microbiol* **1998,** *36*, 1090–1095
3. Kocan, A.A.; Levesque, G.C.; Whitworth, L.C.; Murphy, G.L.; Ewing, S.A.; Barker, R.W. Naturally occurring *Ehrlichia chaffeensis* infection in coyotes from Oklahoma. *Emerg Infect Dis* **2000**, *6*, 477. DOI: 10.3201/eid0605.000505
4. Berri, M.; Rekiki, A.; Boumedine, K.S.; Rodolakis, A. Simultaneous differential detection of *Chlamydophila abortus*, *Chlamydophila pecorum* and *Coxiella burnetii* from aborted ruminant's clinical samples using multiplex PCR. *BMC microbiol* **2009**, *9*, 130. DOI: 10.1186/1471-2180-9-130
5. Milutinović, M.; Masuzawa, T.; Tomanović, S.; Radulović, Ž.; Fukui, T.; Okamoto, Y. *Borrelia burgdorferi* sensu lato, *Anaplasma phagocytophilum*, *Francisella tularensis* and their co-infections in host-seeking *Ixodes ricinus* ticks collected in Serbia. *Exp Appl Acarol* **2008**, *45*, 171–183. DOI: 10.1007/s10493-008-9166-6
6. Relman, D.A.; Falkow, S.; Lepp, P.W.; Schmidt, T.M. The causative agent of bacillary angiomatosis is closely related to *Bartonella bacilliformis*. In Programs and abstracts of the 31st Interscience Conference on Antimicrobial Agents and Chemotherapy. Chicago, 1991; Abstract Number 443
